# Prediction of mechanical ventilation greater than 24 hours in critically ill obstetric patients: ten years of data from a tertiary teaching hospital in mainland China

**DOI:** 10.1186/s12884-020-03524-4

**Published:** 2021-01-09

**Authors:** Huiying Zhao, Guangjie Wang, Jie Lyu, Xiaohong Zhang, Youzhong An

**Affiliations:** 1 Department of Critical Care Medicine, Peking University People’s Hospital, No. 11 Xizhimen South Street, Xicheng District, Beijing, 100044 China; 2 Department of Obstetrics, Peking University People’s Hospital, No. 11 Xizhimen South Street, Xicheng District, Beijing, 100044 China

**Keywords:** Critically ill obstetric patients, Prolonged mechanical ventilation, Risk factors

## Abstract

**Background:**

Maternal admission to the intensive care unit (ICU) during pregnancy or in the postpartum period is a marker of severe acute maternal morbidity. Mechanical ventilation is an important and basic method of maintaining life support in the ICU, but prolonged mechanical ventilation (PMV) is associated with a prolonged length of hospital stay and other adverse outcomes. Therefore, we conducted this retrospective study to describe morbidity and further try to identify the risk factors for PMV in critically ill obstetric women.

**Methods:**

The clinical data were obtained from a single-centre retrospective comparative study of 143 critically ill obstetric patients at a tertiary teaching hospital in mainland China between January 1, 2009, and December 31, 2019. PMV was defined as a mechanical ventilation length of more than 24 h. Clinical and obstetric parameters were collected to analyse the risk factors for PMV. Patients were separated into groups with and without PMV. Potential risk factors were identified by univariate testing. Multivariate logistic regression was used to evaluate independent predictors of PMV.

**Results:**

Out of 29,236 hospital deliveries, 265 critically ill obstetric patients entered the ICU. One hundred forty-five (54.7%) of them were treated with mechanical ventilation. Two were excluded because of death within 24 h. Sixty-five critically ill obstetric patients (45.5%) underwent PMV. The independent risk factors for PMV included estimated blood loss (odds ratio (OR) =1.296, *P*=0.029), acute kidney injury (AKI) (OR=4.305, *P*=0.013), myocardial injury (OR=4.586, *P*=0.012), and PaO_2_/FiO_2_ (OR=0.989, *P*< 0.001). The area under the receiver operating characteristic (ROC) curve based on the predicted probability of the logistic regression was 0.934.

**Conclusions:**

Estimated blood loss, AKI, myocardial injury, and PaO_2_/FiO_2_ were independent risk factors for PMV in critically ill obstetric patients.

**Supplementary Information:**

The online version contains supplementary material available at 10.1186/s12884-020-03524-4.

## Background

Although maternal deaths in China have decreased substantially in recent decades, the incidence of severe acute maternal morbidity (SAMM) is still high [1, 2]. Due to the implementation of the two-child policy in China, since 2016, the number of pregnant women with an older maternal age and history of caesarean section has increased, which has further increased the incidence of gestational diabetes mellitus, dangerous placenta previa, placental implantation, and severe postpartum haemorrhage [3–5]. Maternal admission to the intensive care unit (ICU) during pregnancy or in the postpartum period is a marker of SAMM [2, 6–11]. ICU admission of pregnant and postpartum women presents significant challenges to ICU clinicians because of altered maternal physiology, fetal considerations and medical emergencies associated with pregnancy [12, 13]. Mechanical ventilation is an important and basic manner of maintaining life support in the ICU, but prolonged mechanical ventilation (PMV) increases the risk of a prolonged length of hospital stay, increased hospitalization costs, and other adverse outcomes [14, 15]. Therefore, exploring the risk factors for prolonged mechanical ventilation in critically ill pregnant and postpartum women is important for quality and prognosis improvement. However, little is known about the proportion and risk factors for PMV in critically ill obstetric patients. Therefore, we conducted this retrospective study to describe morbidity and further try to identify the risk factors for PMV in critically ill obstetric women.

## Methods

### Participants

The clinical data were obtained from a single-centre retrospective comparative study of 143 critically ill obstetric patients at a tertiary teaching hospital in mainland China between January 1, 2009, and December 31, 2019. The protocol for this study was approved by the institutional independent Ethics Committee.

The inclusion criteria for this study were obstetric patients (i.e., those who were pregnant or up to 42 days postpartum) with the following conditions: (1) entered the intensive care unit (ICU) and (2) treated with mechanical ventilation (MV). Obstetric patients who met the following criteria would be transferred to the ICU: patients requiring or likely to require advanced respiratory support, patients requiring support of two or more organ systems, and patients with chronic impairment of one or more organ systems who also require support for an acute reversible failure of another organ. The exclusion criteria were as follows: (1) age < 18 years; (2) those who signed do-not-resuscitate; and (3) death within 24 h of the commencement of MV.

### Baseline characteristics

The baseline clinical and obstetric patient characteristics were collected within the first 24 h after mechanical ventilation (the worst results within the first 24 h were chosen) (see Table 1). Clinical parameters included age, causes for ICU admission, Acute Physiology and Chronic Health Evaluation (APACHE) II score, body mass index (BMI), creatinine, acute kidney injury (AKI), troponin T, myocardial injury, total bilirubin (TBIL), albumin, brain natriuretic peptide (BNP), white blood cell (WBC) count, platelets arterial blood pH, arterial partial pressure of carbon dioxide (PaCO_2_), the ratio of the arterial partial pressure of oxygen and the fraction of inspired oxygen (PaO_2_/FiO_2_), and lactate. Acute kidney injury (AKI) was identified according to the Kidney Disease: Improving Global Outcomes (KDIGO) definition as one of the following: 1) An increase in serum creatinine by ≥0.3 mg/dl (≥26.5 μmol/l) within 48 h; 2) An increase in serum creatinine to ≥1.5 times baseline within the previous 7 days; 3) Urine volume ≤0.5 ml/kg/h for 6 h [16]. Myocardial injury was defined as an increase in blood cardiac troponin I with a cut-off value of 34 pg/mL in our hospital [17].

**Table 1:** Comparison of the clinical characteristics of the prolonged mechanical ventilation group and the non-prolonged mechanical ventilation group

Variables	Median [IQR] / no. (%)			
	Overall	PMV group	Non-PMV group	*P* value
Number	143	65	78	
Age, years	31 (28–36)	31 (28–34)	31.5 (28–36)	0.226
BMI, kg/m^2^	27.8 (24.3–30.1)	27.0 (23.9–29.4)	28.3 (25.4–30.4)	0.178
Gestational weeks, weeks	34.9 (30.4–38)	34.2 (29.2–37.2)	35 (32–38)	0.157
APACHE II score	15 (14–18)	16 (14–20)	15 (13–18)	0.008
Causes of the admission of ICU, obstetric causes	90 (62.9%)	46 (70.8%)	44 (56.4%)	0.077
Estimated blood loss, L	1.1 (0.4–3.9)	2.8 (0.7–6.3)	0.7 (0.3–2.4)	< 0.001
Creatinine, μmol/L	60 (44–93)	88 (56–127)	51 (41–64)	< 0.001
AKI	63 (44.1%)	48 (73.9%)	15 (19.2%)	< 0.001
Troponin I, pg/mL	27 (4–183)	106 (32–321)	6 (2–30)	< 0.001
Myocardial injury	61 (42.7%)	47 (72.3%)	14 (18.0%)	< 0.001
TBIL, μmol/L	14.8 (9.5–27.4)	20.2 (11.1–39.2)	12.4 (8.3–22.6)	< 0.001
Albumin, g/L	26.3 (23–29.3)	25.3 (22.6–28.8)	26.7 (23.8–29.9)	0.121
BNP, pg/mL	368 (135–631)	539 (368–820)	194 (91–397)	< 0.001
WBC, × 10^9^/L	13.6 (10.1–16.2)	13.2 (9.7–16.1)	13.7 (10.7–16.2)	0.557
Platelet, × 10^12^/L	74 (40–119)	66 (37–97)	88.5 (53.1–138)	0.026
pH	7.4 (7.4–7.5)	7.4 (7.4–7.5)	7.4 (7.4–7.5)	0.406
PaCO_2_, mmHg	31.9 (27.4–35)	33.3 (28.6–36.8)	30.6 (27–34)	0.008
Bicarbonate, mmol/L	22.3 (19.7–24.1)	23.1 (20.7–25.2)	21.2 (19.2–24.1)	0.071
PaO_2_/FiO_2_, mmHg	322.3 (223.5–441)	240 (173.3–318.3)	403.4 (319.8–501.5)	< 0.001
Lactate, mmol/L	1.4 (0.9–2.6)	1.8 (1.1–3.4)	1.2 (0.8–1.9)	0.002

Abbreviations: *IQR* interquartile ranges, *PMV* prolonged mechanical ventilation, *BMI* body mass index, *APACHE* acute physiology and chronic health evaluation, *ICU* intensive care unit, *AKI* acute kidney injury, *BNP* brain natriuretic peptide, *WBC* white blood cell, *TBIL* total bilirubin, *no.* Number, *PaCO*_*2*_ arterial partial pressure of carbon dioxide, *PaO*_*2*_*/FiO*_*2*_ the ratio of the arterial partial pressure of oxygen and the fraction of inspired oxygen

Obstetric parameters included gestational weeks, obstetric causes of ICU admission, and the estimated blood loss volume during delivery.

### Clinical outcomes

The outcomes of the critically ill obstetric patients in our study included the length of ICU stay, length of hospital stay, length of mechanical ventilation, and hospital mortality.

### Prolonged mechanical ventilation

Previous studies used predefined values of mechanical ventilation time, ranging from 24 h to 72 h, and even to 21 days, to define critically ill patients as having PMV [18]. In this study, PMV was defined as a mechanical ventilation length of more than 24 h. This definition was in accordance with some previous studies [19, 20] and also guided by the median length of mechanical ventilation of this study. According to PMV status, patients were divided into two groups: the PMV group and the non-PMV group. The ventilation principles and weaning protocol were listed in the additional files (see Additional file 2).

### Statistical analysis

We evaluated the normality of the continuous variables and found that all data were abnormally distributed. Therefore, continuous data were expressed as medians with interquartile ranges. Categorical variables were expressed as proportions. In the univariate testing, continuous and categorical variables were examined using Kruskal-Wallis equality-of-populations rank tests or Pearson chi-square tests, respectively. Variables with a significance of less than 0.05 on univariate analysis were identified as risk factors. We used the variance inflation factor (VIF) and tolerance to test the multicollinearity of the risk factors. A VIF more than 10 or tolerance less than 0.1 was identified as significant multicollinearity. Independent risk factors for PMV was determined by binary logistic regression. We used Hosmer-Lemeshow test to estimate the goodness of fit. We used predictive probability values from logistic regression to generate the receiver operating characteristic (ROC) curve. And the regression coefficients were plotted by the coefplot module. We used a nomogram to demonstrate the risk points and probability for predicting PMV. A *P* value of less than 0.05 in 2-sided tests was the threshold for statistically significant. Analyses were performed with Stata software, version 15.1 (Stata Corp).

## Results

### Participants

**Fig. 1:**
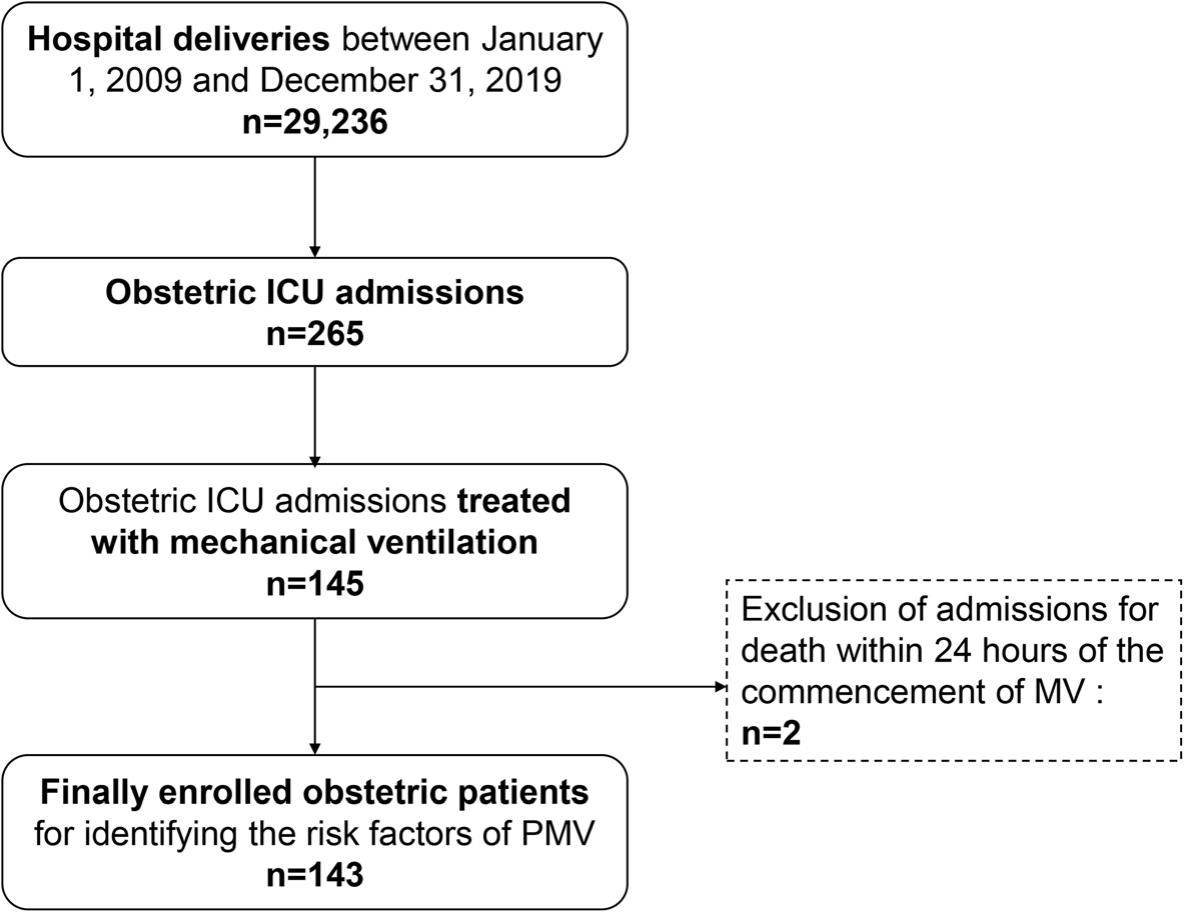
Algorithm for selection of critically ill obstetric patients with mechanical ventilation

Out of 29,236 hospital deliveries, 265 critically ill obstetric patients entered the ICU between January 1, 2009, and December 31, 2019, in our hospital. One hundred forty-five (54.7%) of them were treated with mechanical ventilation. Two were excluded because of death within 24 h of the commencement of MV. Finally, one hundred forty-three patients were enrolled in this study (see Fig. 1). Among these patients, 52 (36.4%) were transferred from other hospital and all of them gave birth at our hospital. Seven (4.9%) of those patients were admitted during pregnancy and 136 (95.1%) admitted postpartum.

The median age was 31 years, and the median gestational week was 34.9 weeks. Obstetric causes made up the majority of the causes of ICU admission (62.9%, 90/143). The main obstetric cause was postpartum haemorrhage (35%, 50/143). Other obstetric causes included eclampsia or pre-eclampsia (18.9%, 27/143), puerperal infection (5.6%, 8/143), acute fatty liver of pregnancy (AFLP) (2.1%, 3/143) and others (1.4%, 2/143). Nonobstetric causes included haematological diseases (11.2%, 16/143), autoimmune diseases (5.6%,8/143), cardiovascular disease (2.1%, 3/143), neuropsychiatric diseases (4.2%, 6/143), acute pancreatitis (3.5%, 5/143), gastrointestinal perforation (3.5%, 5/143) and others (7.0%, 10/143). The median length of mechanical ventilation was 19 h. The median length of ICU stay and hospital stay were 72 h and 13 days, respectively. Four obstetric patients died in the hospital (2.8%) (see Tables 1 and 2).

**Table 2:** Comparison of outcomes for the prolonged mechanical ventilation group and the non-prolonged mechanical ventilation group

Outcomes	Median [IQR] / no. (%)			
	Overall	PMV group	Non-PMV group	*P* value
Number	143	65	78	
Length of ICU stay, hours	72 (27–168)	168 (99–312)	31 (23–67)	< 0.001
Length of hospital stay, days	13 (9–20)	15 (12–26)	11 (8–16)	< 0.001
Mortality	4 (2.8%)	3 (4.6%)	1 (1.3%)	0.229

Abbreviation: *IQR* interquartile ranges, *ICU* intensive care unit, *PMV* prolonged mechanical ventilation

### Prolonged mechanical ventilation and outcomes

Sixty-five critically ill obstetric patients (45.5%) underwent PMV. The length of ICU stay (*P*< 0.001) and hospital stay (*P*< 0.001) were both significantly longer in the PMV group than in the non-PMV group. The mortality was very low, and there was no significant difference between the two groups (*P*=0.229).

### Univariate analysis

Compared with non-PMV patients, PMV patients had a higher APACHE II score (*P*=0.008) and a larger amount of estimated blood loss during delivery (*P*< 0.001). The incidence of AKI (*P*< 0.001) and myocardial injury (*P*< 0.001) was significantly higher in the PMV group than in the non-PMV group. PMV patients had significantly higher blood creatinine(*P*< 0.001), troponin(*P*< 0.001), TBIL (*P*< 0.001), BNP (*P*< 0.001), PaCO_2_ (*P*=0.012), and lactate (*P*=0.002) levels. The blood platelet count (*P*=0.026) and the arterial blood PaO_2_/FiO_2_ (*P*< 0.001) were significantly lower in the PMV group. No significant difference in age, BMI, gestational weeks, causes of ICU admission, blood WBC count, blood albumin level, arterial blood pH, or bicarbonate was observed (see Tables 1 and 2).

### Multivariate analysis

**Fig. 2:**
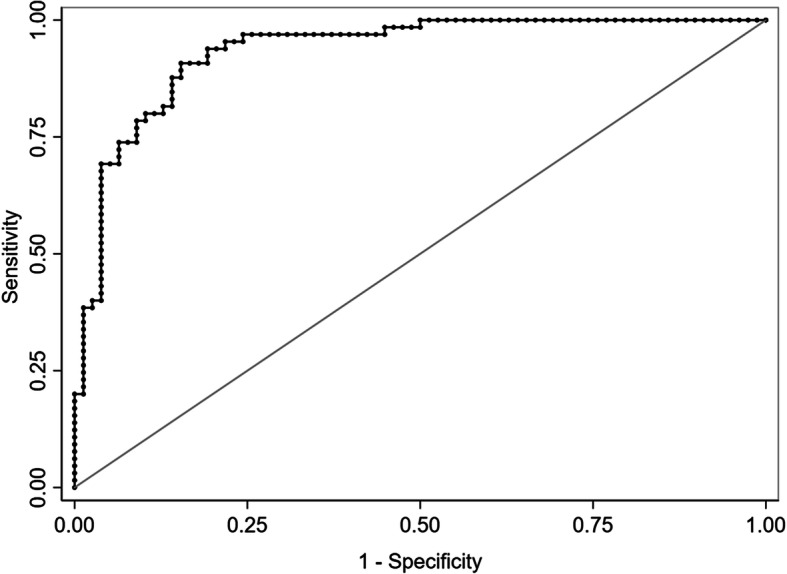
The ROC curve using predicted probability values from the logistic regression. The area under the curve was 0.934, 95% CI was 0.895 to 0.974

**Fig. 3:**
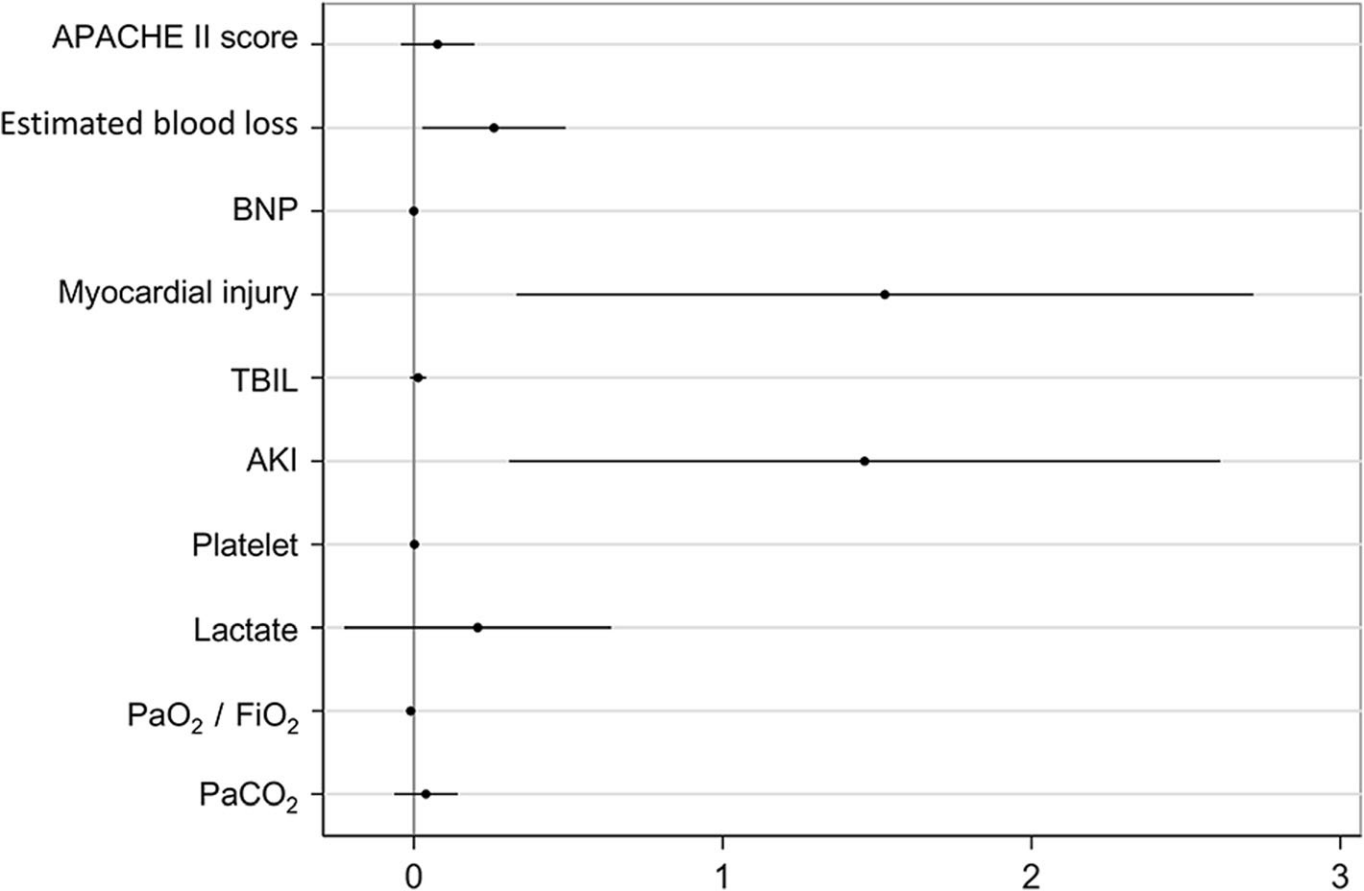
Coefplot of the Logistic regression coefficient. Estimated blood loss, AKI, and myocardial injury were independent risk factors for PMV, but PaO_2_/FiO_2_ was independent protective factor

Variables including APACHE II score, BMI, AKI, myocardial injury, TBIL, BNP, platelets, PaCO_2_, PaO_2_/FiO_2_, and estimated blood loss with a *P* value of < 0.05 in the univariate analysis were identified as risk factors for PMV. We tested the multicollinearity of all the risk factors. The results showed that the largest VIF was 1.66 and the smallest tolerance was 0.60 (see Additional file 1). Therefore, we included all 10 chosen risk factors in the logistic regression model, and we found that estimated blood loss (L) (odds ratio (OR) =1.296, *P*=0.029), AKI (OR=4.305, *P*=0.013), myocardial injury (OR=4.586, *P*=0.012), and PaO_2_/FiO_2_ (mmHg) (OR=0.989, *P*< 0.001) were independent risk factors for PMV in critically ill obstetric patients (see Table 3). The Hosmer-Lemeshow test showed that the fit for the logistic regression model was good (*P*=0.097, chi2=153.56). The ROC curve based on the predicted probability of the logistic regression is shown in Fig. 2, and the area under the curve was 0.934 (95% CI, 0.895 to 0.974). The plot of the regression coefficients is shown in Fig. 3.

**Table 3:** Logistic regression of prolonged mechanical ventilation and clinical variables

Variables	OR	*P* value	95% CI
Estimated blood loss, L	1.296	0.029	1.028–1.634
Myocardial injury	4.596	0.012	1.394–15.159
AKI	4.305	0.013	1.361–13.617
PaO_2_/FiO_2_, mmHg	0.989	< 0.001	0.984–0.995
APACHE II score	1.080	0.207	0.958–1.216
BNP, pg/mL	0.999	0.399	0.999–1.000
TBIL, μmol/L	1.014	0.317	0.987–1.041
Platelet, × 10^12^/L	1.002	0.647	0.995–1.009
Lactate, mmol/L	1.229	0.349	0.798–1.894
PaCO_2_, mmHg	1.040	0.457	0.938–1.152

Abbreviations: *APACHE* acute physiology and chronic health evaluation, *AKI* acute kidney injury, *BNP* brain natriuretic peptide, *TBIL* total bilirubin, *PaCO*_*2*_ arterial partial pressure of carbon dioxide, *PaO*_*2*_*/FiO*_*2*_ the ratio of the arterial partial pressure of oxygen and the fraction of inspired oxygen, *OR* odds ratio, *CI* confidence interval

### Nomogram

**Fig. 4:**
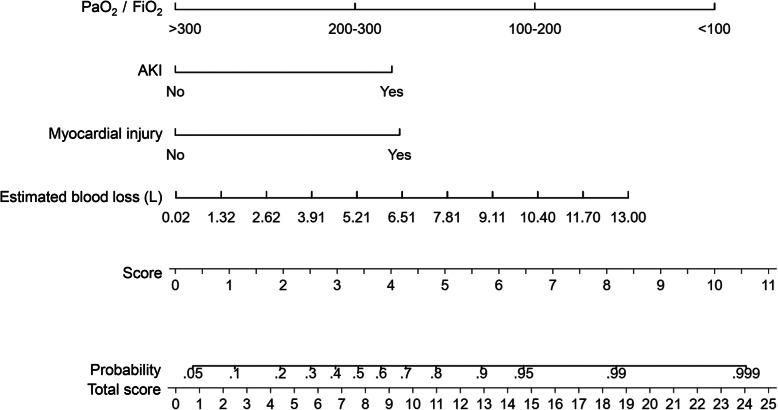
Nomogram for prolonged mechanical ventilation using the independent prognostic factors critically ill obstetric patients. For example: If a patient was with AKI, myocardial injury, estimated blood loss of 3.91 L, and PaO_2_/FiO_2_ of 250 mmHg. The scores were 4, 4, 2.5 and 3.5, respectively. Then the total score was 14 and the probability of PMV was approximately 93%

Through the logistic regression model, we built a prognostic nomogram incorporating the above independent prognostic factors for visualization and facilitation of clinical practice, as shown in Fig. 4. In this model, we transferred PaO_2_/FiO_2_ into four classes based on the Berlin definition of acute respiratory distress syndrome (ARDS) to simplify clinical practice [21].

## Discussion

To our knowledge, this is the first study to identify the characteristics of mechanical ventilation and the risk factors for PMV in critically ill pregnant and postpartum patients. In our study, the incidence of mechanical ventilation was 54.7% among obstetric patients admitted to the ICU. Estimated blood loss, AKI, myocardial injury, and PaO_2_/FiO_2_ were independent risk factors for PMV in critically ill obstetric patients.

In our study, more than half of critically ill obstetric patients received mechanical ventilation, and 45.5% of them had a ventilation time of more than 24 h*.* These results are similar to those of another multicentre report. Zhao et al. enrolled 491 obstetric patients with ICU admissions from three tertiary hospitals in China. They found that 40% of the patients underwent intubation and mechanical ventilation, and the median length of mechanical ventilation was 1 day [2].

Our study showed that estimated blood loss was an independent risk factor for the development of PMV. Several studies have shown that the most common cause of pregnancy-related admission to the ICU is obstetric haemorrhage [2, 12]. A multicentre study in China found that postpartum haemorrhage (170/491; 34.62%) was the main reason for ICU admission [2]. Chantry et al. determined the reasons for pregnancy-related ICU admissions from all ICUs in France from 2006 to 2009. They enrolled 11,824 pregnancy-related ICU admissions and showed that the leading cause of transfer to ICUs was obstetric haemorrhage (4043; 34.2%) [12]. We also found that postpartum haemorrhage was the main reason for ICU admission (87/143; 35%). Possible reasons for the association of estimated blood loss and PMV included the following: massive obstetric haemorrhage that induced hypovolemic shock and tissue hypoperfusion and further caused lung injury; an independent, dose-dependent relationship between blood transfusion and the subsequent development of acute lung injury; and massive haemorrhage and subsequent resuscitation leading to fluid overload, which is associated with pulmonary oedema and ventilator dependence [22, 23]. Surgical procedures for hemostasis including uterine artery ligation, uterine packing, and hysterectomy are usually used for managing the massive hemorrhage.

In our study, obstetric patients with an AKI were more likely to have PMV than those without an AKI. Ghauri et al. systematically reviewed the predictors of the need for PMV in adult patients admitted to ICUs for medical and surgical needs. They found that kidney dysfunction was one of the most significant independent predictors of the need for PMV [18]. Clark et al. retrospectively assessed 130 ICU patients and showed that serum creatinine levels greater than 2.0 mg/dL were independently associated with PMV. They further validated their findings in a prospective trial. Acute kidney injury is one of the manifestations of insufficient organ perfusion [24]. On the other hand, kidney dysfunction can cause fluid accumulation and tissue oedema. Therefore, it is very important to monitor renal function and treat potential causes in critically ill obstetric patients in a timely manner.

Our results demonstrated that myocardial injury was an independent risk factor for the development of PMV. We defined myocardial injury as an increase in blood cardiac troponin I [17]. Myocardial injury may be caused by hypoperfusion, hypoxia, global ischaemia, surgery, and sepsis in noncardiac patients. Karpati et al. enrolled 55 parturients with severe postpartum haemorrhage and haemorrhagic shock and found that 51% of them had myocardial injury (elevated serum levels of cardiac troponin I). They also identified low systolic and diastolic arterial blood pressure (< 88 and < 50 mmHg, respectively) and increased heart rate (> 115 beats/min) as independent predictors of myocardial injury [25]. A systematic review included nine studies involving 719 patients who suffer from pre-eclampsia and concluded that in such pre-eclampsia pregnant women cardiac troponin I might be elevated [26]. Previous studies also showed that myocardial injury was associated with mortality among patients undergoing noncardiac surgery [17, 27, 28]. Our previous study retrospectively enrolled 381 critically ill patients who underwent major abdominal surgery. We found that myocardial injury is an independent risk factor for weaning failure from mechanical ventilation [29]. Abdalla et al. also demonstrated that troponin elevation was associated with a longer duration of mechanical ventilation in patients who were admitted to the ICU with sepsis [27].

According to our logistic regression analysis, PaO_2_/FiO_2_ is an independent risk factor for the development of PMV. PaO_2_/FiO_2_ is an important variable for evaluating lung injury, and it is the basis for the Berlin definition of ARDS risk stratification [21]. PaO_2_/FiO_2_ is also an important indicator for evaluating mechanical ventilation weaning [30]. Therefore, we need to monitor PaO_2_/FiO_2_ to evaluate the lung function of critically ill obstetric patients and improve PaO_2_/FiO_2_ through mechanical ventilation and other aetiological treatments.

We found that not only the pulmonary function parameter PaO_2_/FiO_2_ was a risk factor for PMV, but also estimated blood loss, AKI, and myocardial injury were independent risk factors. Therefore, the following measures are crucially important in clinical practice so as to shorten the length of mechanical ventilation: 1) early recognize and timely control the obstetric haemorrhage; 2) maintain hemodynamic stability and tissue perfusion to prevent organ dysfunction; 3) closely monitor the function of kidney, heart, and other organs in critically ill obstetric patients; 4) use lung-protective ventilation in lung injury patients (PaO_2_/FiO_2_< 300 mmHg). In summary, the pre-ICU admission phase is very important and worthy of further research to shorten the mechanical ventilation time of critically ill obstetric patients.

This study has several limitations. First, the data were retrospectively collected. We could not control for some variables, which may have resulted in data bias. Second, the study was a single-centre study, and most obstetric patients were admitted to the ICU based on local criteria and local practice. Third, we did not distinguish the patients between pregnant and postpartum phase. Fourth, we did not analysis the data or the potential influence of transfusion of blood products. Fifth, most of the clinical parameters we included were collected after admission to ICU, so this study may have a delay in recognition of clinical deterioration. Sixth, we derived four independent risk factors for prolonged mechanical ventilation and built a nomogram for visualization and facilitation of clinical practice. However, we did not validate the nomogram with a new database. A prospective, multicentre study is needed to address these issues and validate our findings.

## Conclusions

This retrospective study shows that the independent risk factors for PMV in critically ill obstetric patients are estimated blood loss, AKI, myocardial injury, and PaO_2_/FiO_2_. The pre-ICU admission phase is worthy of further research to shorten the mechanical ventilation time of critically ill obstetric patients.

## Supplementary Information

**Additional file 1.** Multicollinearity test of risk factors for prolonged mechanical ventilation.

**Additional file 2.** Ventilation principles. Ventilator Weaning Protocol.

## Data Availability

The authors declare that all data supporting the findings of this study are available within the article and its additional Files.

## Abbreviations

AFLP: Acute fatty liver of pregnancy
AKI: Acute kidney injury
APACHE: Acute physiology and chronic health evaluation
ARDS: Acute respiratory distress syndrome
BMI: Body mass index
BNP: Brain natriuretic peptide
CI: Confidence interval
ICU: Intensive care unit
IQR: Interquartile ranges
KDIGO: Kidney Disease: Improving Global Outcomes
MV: Mechanical ventilation
OR: Odds ratio
PaCO_2_: Arterial partial pressure of carbon dioxide
PaO_2_/FiO_2_: Ratio of arterial partial pressure of oxygen and fraction of inspired oxygen
PMV: Prolonged mechanical ventilation
ROC: Receiver operating characteristic
SAMM: Severe acute maternal morbidity
TBIL: Total bilirubin (TBIL)
WBC: Blood white blood cell
VIF: Variance inflation factor
