# Supplementary material for: Prediction of mechanical ventilation greater than 24 hours in critically ill obstetric patients: ten years of data from a tertiary teaching hospital in mainland China
Source: BMC Pregnancy Childbirth. 2021 Jan 9;21:40. doi: 10.1186/s12884-020-03524-4 (PMC7796589; doi:10.1186/s12884-020-03524-4)
Supplement: Supplementary file 1 — Additional file 1. Multicollinearity test of risk factors for prolonged mechanical ventilation. [file 12884_2020_3524_MOESM1_ESM.docx]

**Additional file 1** Multicollinearity test of risk factors for prolonged mechanical ventilation

| Risk factors | VIF | Tolerance |
| --- | --- | --- |
| APACHE II score | 1.15 | 0.87 |
| Estimated blood loss | 1.42 | 0.70 |
| AKI | 1.63 | 0.62 |
| myocardial injury | 1.66 | 0.60 |
| TBIL | 1.16 | 0.86 |
| BNP | 1.39 | 0.72 |
| Platelet | 1.08 | 0.93 |
| PaO_2_/FiO_2_ | 1.48 | 0.68 |
| Lactate | 1.38 | 0.72 |

Abbreviations: APACHE acute physiology and chronic health evaluation, AKI acute kidney injury, BNP brain natriuretic peptide, TBIL total bilirubin, PaO_2_/FiO_2_ the ratio of the arterial partial pressure of oxygen and the fraction of inspired oxygen, VIF variance inflation factor.
