# Supplementary material for: Prediction of mechanical ventilation greater than 24 hours in critically ill obstetric patients: ten years of data from a tertiary teaching hospital in mainland China
Source: BMC Pregnancy Childbirth. 2021 Jan 9;21:40. doi: 10.1186/s12884-020-03524-4 (PMC7796589; doi:10.1186/s12884-020-03524-4)
Supplement: Supplementary file 2 — Additional file 2. Ventilation principles. Ventilator Weaning Protocol. [file 12884_2020_3524_MOESM2_ESM.docx]

**Additional file 2**

**Ventilation principles**

1. Ventilation principles for critically ill obstetric patients without acute respiratory distress syndrome (ARDS)

1) Tidal volume (Vt): 6-8mL/kg (predicted body weight)

2) P_plat_≤30 cmH_2_O

3) Respiratory rate (RR): 12-20 bpm

4) PEEP: 5-8cmH_2_O

5) FiO_2_: 40-60%

6) Duration of inspiration / duration of expiration: 1:1.5-2.5

2. Ventilation principles for critically ill obstetric patients who was diagnosed as ARDS.

1) Vt: 4-8mL/kg (predicted body weight).

2) P_plat_≤30 cmH_2_O.

3) Initial respiratory rate: 12-20 bpm.

4) Oxygenation goal: PaO_2_ 55-80 mmHg or SpO_2_ 88-95%.

Use incremental FiO_2_/PEEP combinations shown below to achieve goal.

| FiO_2_ | 0.3 | 0.4 | 0.4 | 0.5 | 0.5 | 0.6 | 0.7 | 0.7 | 0.7 | 0.8 | 0.9 | 0.9 | 0.9 | 1.0 |
| --- | --- | --- | --- | --- | --- | --- | --- | --- | --- | --- | --- | --- | --- | --- |
| PEEP | 5 | 5 | 8 | 8 | 10 | 10 | 10 | 12 | 14 | 14 | 14 | 16 | 18 | 18-24 |

5) pH goal: 7.30-7.45

If pH 7.15-7.30: increase RR until pH>7.30 or PaCO2<25 (Maximum set RR=35).

If pH <7.15: increase RR to35. If pH remains <7.15, Vt may be increased in 1 mL/kg steps until pH>7.15 (P_plat_ target of 30 cmH_2_O may be exceeded).

**Ventilator Weaning Protocol**

1. Conduct a spontaneous breathing trial (SBT) daily when:

1. Underlying reason for intubation is reversed or improving.
2. Difficult intubations/airway will be noted in ICU team discussions prior to SBT.
3. Intact airway reflexes.
4. Hemodynamics are stable, no significant dysrhythmias, ischemia, or high dose inotropes.
5. Minute ventilation less than 20 L/min Fluid, electrolyte, and acid-base status are appropriate.
6. FiO_2_ 50% or less and PEEP less than or equal to 8. PaO2/FiO2 greater than or equal to 150 mmHg.
7. The patient is without neuromuscular blockade.

2. SBT Procedure

Patient is placed on Pressure Support 5-8 cmH2O, and PEEP 5 cmH2O. 30-120min

3. SBT Failure – if any of the following measurements are maintained, document SBT failure and place the patient back on previous ventilator settings. Document the reason for SBT failure. If all of the following measurements are not encountered, document SBT success.

1. RSBI is 105 or greater.
2. Ve greater than 20 L.
3. Vt less than 4 ml/kg IBW.
4. SpO_2_ less than 90 % saturation.
5. Systolic Blood Pressure greater than 180 or less than 90 mmHg.
6. Respiratory Distress. RR>30
7. HR greater than 120 % of baseline HR or greater than 140 bpm or less than 50 bpm, or a change of ± 20% (less than 5 min of increased HR may be tolerated).
8. Marked use of accessory muscles; Abdominal paradox; Diaphoresis; Marked subjective dyspnea.
9. Apnea.
